# GCAT: A network model of mutational influences between amino acid positions in PSD95^pdz3^

**DOI:** 10.3389/fmolb.2022.1035248

**Published:** 2022-10-31

**Authors:** Lorenza Pacini, Claire Lesieur

**Affiliations:** ^1^ University Lyon, CNRS, INSA Lyon, Ecole Centrale de Lyon, UMR5005, Université Claude Bernard Lyon 1, Villeurbanne, France; ^2^ Institut Rhônalpin des Systèmes Complexes, IXXI-ENS-Lyon, Lyon, France

**Keywords:** proteins, network-based models, mutations, protein sustainable design, PSD95^pdz3^

## Abstract

Proteins exist for more than 3 billion years: proof of a sustainable design. They have mechanisms coping with internal perturbations (e.g., amino acid mutations), which tie genetic backgrounds to diseases or drug therapy failure. One difficulty to grasp these mechanisms is the asymmetry of amino acid mutational impact: a mutation at position *i* in the sequence, which impact a position *j* does not imply that the mutation at position *j* impacts the position *i*. Thus, to distinguish the influence of the mutation of *i* on *j* from the influence of the mutation of *j* on *i*, position mutational influences must be represented with directions. Using the X ray structure of the third PDZ domain of PDS-95 (Protein Data Bank 1BE9) and *in silico* mutations, we build a directed network called GCAT that models position mutational influences. In the GCAT, a position is a node with edges that leave the node (out-edges) for the influences of the mutation of the position on other positions and edges that enter the position (in-edges) for the influences of the mutation of other positions on the position. 1BE9 positions split into four influence categories called G, C, A and T going from positions influencing on average less other positions and influenced on average by less other positions (category C) to positions influencing on average more others positions and influenced on average by more other positions (category T). The four categories depict position neighborhoods in the protein structure with different tolerance to mutations.

## Introduction

Proteins exist for more than 3 billion years, testimony of a sustainable design that allows them to cope with internal perturbations (amino acid mutations) and external perturbations (environmental conditions) through time (Woolfson et al., 2012; Parisi et al., 2015). The protein sustainable design is one key to link individual genetic backgrounds to individual health and drug-therapy efficiency because genetic backgrounds modulate protein sequences (sequence variants, mutations), which in turn modulate the biological function of proteins which if faulty can lead to diseases.

There are three functional consequences to mutations: 1) functional reproducibility (robustness), 2) functional innovation (adaptation) and 3) functional failure (e.g., pathologies). If the mutation of an amino acid type *a* to an amino acid type *b* at a position *i* in the protein sequence leads to functional innovation or functional failure, it means that the changes due to the mutation at position *i* functionally influence the system. Here the system consists of the other {*j}* amino acid positions in the protein sequence. On the contrary, if the same mutation from type *a* to type *b* at position *i* leads to functional reproducibility, it means that the changes due to the mutation at position *i* have no functional influence on the system: the protein sequence with type *a* at position *i* and the protein sequence with type *b* at position *i* are functional alternatives. In other words, in the case of functional reproducibility the function is still encoded despite one sequence-error, contributing to the system sustainability.

Now, amino acid mutations can influence the protein structure and/or its dynamics prior influencing its function such that understanding how proteins cope with mutations requires to monitor changes not only on the function but on the structure and the dynamics. From there, the issue is to distinguish changes tolerated by the structure which yield structural alternative and structurally robust solutions from changes not tolerated, which identify potential needs for “corrections” to accommodate the mutations. Likewise, this applies to dynamical and functional changes. Computational biology and learning methods, which can screen large dataset, offer several efficient tools and methods to analyze protein tolerance to mutations, more generally to analyze the mutational fate of amino acid positions (Vuillon and Lesieur, 2015; Ponzoni and Bahar, 2018; Poelwijk et al., 2019; Liang et al., 2020; Pacini et al., 2020; Demir et al., 2021; Pacini et al., 2021; Wingert et al., 2021; Xavier et al., 2021; Sen et al., 2022).

The features of amino acid positions (e.g., rASA, amino acid types, secondary structures, *etc.*) are involved in the mutational fate of positions as revealed by the higher tolerance to mutations of surface exposed positions (Halabi et al., 2009) or the high frequency of Trp or Cys (involved in disulfide bridge) mutations in diseases (Iqbal et al., 2020). Now Trp and Cys features are rather unique among the twenty most common amino acids, which means reproducing their features with alternative amino acid types is unlikely. That may be why they are intolerant to mutation and often involved in diseases when mutated.

There are also evidences that the mutational fate of a position depends on amino acid types at other positions. Pairwise position interdependency is shown by co-evolution analysis and identification of sector positions (Halabi et al., 2009; McLaughlin et al., 2012). Rescue mechanisms where the impact of individual mutations involved in cancers are prevented by mutations elsewhere are other examples of amino acid pairwise position interdependency (Demir et al., 2011). Functional innovation and adaptation mechanisms where coupled mutations introduce a new function while individually each mutation reproduce the original function is also showing the role of another position in the mutational fate of a position (Amitai et al., 2007). The functional tolerance to double mutations means that the function remains decipherable despite the two errors in the protein sequence, improving the system sustainability compared to a system tolerating only single errors. Moreover, the system is capable of correcting errors by introducing errors elsewhere, improving even more the system sustainability by increasing the diversity of functional sequence alternatives compared to systems with unique functional solutions.

Finally, there are evidences of the role of amino acid neighbors and hence of the influence of more than one other position on a position mutational fate. For example, experimental results show that the mutation of one amino acid type by another type does not systematically have the same effect, indicating that the type at the position is not embedding all the information of the mutational fate, the position neighborhood matters as well. Computational biology also contributes in investigating the impact of multiple mutations on protein fate showing the role of neighborhoods (Pires et al., 2014; Achoch et al., 2016; Dorantes-Gilardi et al., 2018; Liu et al., 2021). In summary, the mutational fate of a position *i* is encoded by a set of amino acid types at the position *i* given a set of *{j}* amino acid type neighbors. Said otherwise, there exist position neighborhoods that encode structural robustness, others that encode structural innovation and yet others, structural failure. Likewise, this applies to dynamics and functions.

Here we propose to investigate the mutational fate of position by classifying position neighborhoods according to their tolerance to mutations and thus identifying positions robust to mutations and positions potentially needing corrections to tolerate mutations. To tackle the complexity related to changes on structure, dynamics and function, we simplify the problem by three means. Firstly, we use *in silico* mutations such that the structural integrity is maintained upon mutations and the problem is now limited to identifying neighborhoods maintaining structural integrity upon mutations from neighborhoods introducing changes and hence potentially requiring corrections to avoid downstream effects. Secondly, we use Fold X so only the side chain at the site of mutation is changed and neighbors are not allowed to move to prevent accommodation of the mutation through motions, deemed a correction. Thirdly, the tolerance of a position to its own mutation and the tolerance of a position to mutations at other positions are considered. This is to take into account the fact that a position may influence positions upon mutation and be influenced by the mutations at other positions.

We developed a directed network, called the GCAT network that models the direction of changes upon mutation with edges directed from *i* to *j* to represent the influence of the mutation at position *i* on other positions *j* (out-edges) and with edges directed from *j* to *i* (in-edges) to represent the influence of the mutations at positions *j* on *i*. Influences are measured by a change in position neighborhoods upon mutations, and the objective is to split positions according to mutation tolerance with one class where the neighborhoods have less than average changes and a class that have more than average changes, considered intolerant and in need of corrections to accommodate the mutations.

Our model of study is the third PDZ domain of the synaptic protein PDS-95 (PSD95pdz3, PDB 1BE9) because the position functional sensitivity is known as well as the sector positions which are coevolving amino acid positions (Halabi et al., 2009; McLaughlin et al., 2012).

The 1BE9 GCAT network is built from the comparison of the neighborhoods of every position in the WT PDB and in the 19N PDB mutants generated with Fold X (N is the number of amino acids in the 1BE9 PDB). The neighborhood information is extracted from each amino acid network built from each PDB (Methods). 1BE9 positions distribute into four categories of influences consistent with the capacity of the protein system to generate changes (G category), connect positions (C category), absorb changes (A category) and transmit changes (T category). Positions in the C and A categories modify on average the neighborhood of less other positions than the positions in the G and T categories and hence the former are considered tolerant to mutations while the latter are not and need adjustment of neighbors to accommodate mutations at the positions.

## Methods

Case of study- The third PDZ domain of the synaptic protein PDS-95 (PSD95pdz3) is our case of study and we use its PDB 1BE9 (Doyle et al., 1996).

rASA- The relative Accessible Surface Area (rASA) of each amino acid of 1BE9 is computed using the DSSP method implemented in BioPython (Cock et al., 2009) and amino acids are classified as surface exposed if rASA >0.2 and buried otherwise. Over the 115 amino acids of PSD95pdz3, 64 are surface exposed (SE) and 51 are buried.

Amino acid network (AAN)- Starting from the Protein Data Bank (PDB) (Berman et al., 2000) data, protein structures are modeled using the Amino Acid Network (AAN) (Dorantes-Gilardi et al., 2018), an established model in Computational Biology. The AAN is a graph *G* = (*V; E*), with *V* the set of the *N* nodes of the network and E the set of links of the network. Nodes and links are also called vertices and edges of the graph, respectively. The edges constitute the pairs of the nodes (*v, w*) connected in the graph. The AAN is an undirected graph where the edges are unordered pairs of nodes.

Nodes of the AAN. Each node in the AAN corresponds to one amino acid of the protein’s sequence:

V={i | i is an amino acid}

Links of the AAN. A link is an atomic interaction defined by atomic proximity: two amino acids *i* and *j* are connected if there exists at least one couple of atoms, one belonging to *i* and one belonging to *j*, at a distance one from the other lower or equal to a given threshold. The threshold is fixed to 5 Å:

E={(i,j) |i,j∈V with i≠jand∃(atomi∈i,atom j∈j) with dist(atomi, atomj)≤5Å}

The AAN is built using Rodrigo Dorantes-Gilardi’s implementation in the Biographs module available at https://github.com/rodogi/biographs.

Node degree. The node degree *k*_*i*_ is defined as the number of neighbors of a node *i*. In the AAN, the node degree corresponds to the number of chemical/first neighbors of a node *i*, i.e., amino acids whose atoms are located at a distance ≤ 5Å of atoms of *i*.

Position neighborhood. The position neighborhood is the list of amino acid neighbors of a position, namely all the neighbors with at least one atom located within ≤ 5Å of at least one atoms of *i*.

*In silico* mutants- All the 19 possible mutations of all the amino acids of a protein are produced *in silico* using FoldX (Schymkowitz et al., 2005) version 5, producing 19 single-amino acid mutants per amino acid position. First, the PDB structure of the protein is repaired using the FoldX command RepairPDB. Then, the *in silico* mutations are performed on the repaired structure using the BuildModel command. All parameters were set at their default values. The RepairPDB procedure is advised in FoldX before making the *in silico* mutation because it makes sure that the WT protein structure on which the mutations are performed does not contain steric clashes, bad torsion angles and side-chains orientations that do not correspond to energy minima. The details can be found in the FoldX documentation (http://foldxsuite.crg.eu/command/RepairPDB).

GCAT network- The GCAT network is built to model the directions of changes caused by *in-silico* amino-acid mutations in a protein structure in the form of a directed network. A directed network is a network where the edges are ordered pairs (*v, w*) of nodes; *v* is the tail and *w* is the head of the edge. In contrast to the AAN, the GCAT network is directed because it models changes on *j* positions when a position *i* is mutated with an edge from *i* to *j* (out-edges) and it models changes on *i* when *j* positions are mutated with an edge going from *j* to *i* (in edges). The mutation of an amino acid *a* at position *i* may change a position *j*, it does not imply that the mutation of *j* changes the position *i,* hence the need of modeling the direction of mutational changes.

**FIGURE 1:**
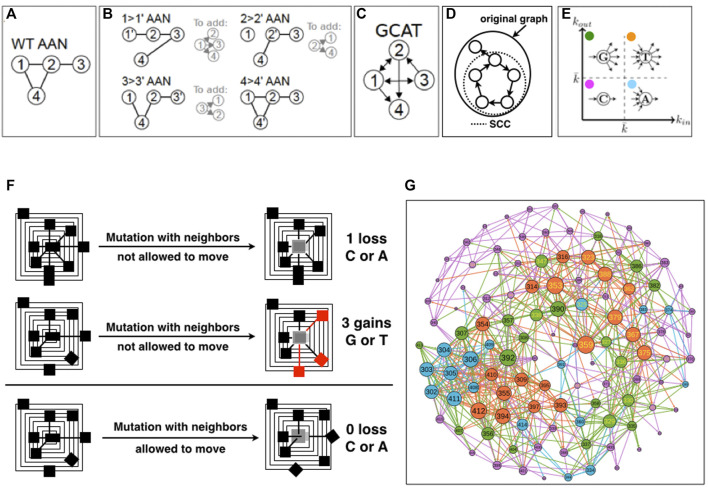
Schematics of the construction of the GCAT network. **(A)**. The Amino Acid Network (AAN) of the wild-type protein is created. Nodes correspond to amino acids and are named by the position in the amino-acid sequence of the protein. **(B)**. The AAN of mutants are created. For each amino acid (i.e., each node of the AAN), the AAN of all its 19 mutants is created. For each mutation i- > i’, if an amino acid j has changed amino-acid neighborhood in the mutant’s AAN compared to the WT AAN, then an edge from the mutation site *i* to *j* is added in the GCAT network. Here, only one mutation per amino acid is represented, for simplicity. **(C)**. The resulting GCAT network. **(D)**. Schematic of the strongly connected component (SCC) of a directed graph. **(E)**. Classification of nodes as G (Generate), C (Connect), A (Absorb) and T (Transmit) in the GCAT network, based on the in- and out-degree. **(F)**. Schematics of neighborhood changes upon *in silico* mutation. **(G)**. The GCAT network of 1BE9.

First, we create all the AANs of the WT protein and of the protein mutants, i.e., the 19 times the length of the amino acid sequence possible single-amino acid mutants with Fold X, and extract the amino acid neighborhoods of all the amino acid positions in the *in-silico* mutants and in the WT structure from their respective AANs. We then compare the neighborhood of each node in each mutant AAN with respect to the WT AAN and use this information to measure a change (Figures 1A–C). If there exists a mutation of *i* that changes the list of neighbors of a node *j* in the mutant AAN with respect to the WT AAN, we say that *i* influences the amino acid *j*. We recall that in the GCAT network, the influence of *i* on *j* is represented by an arc e_ij_ that leaves *i*; we call it an out-edge (*i* → *j*) (Figure 1C, nodes 2 and 3). Now from the point of view of *j*, the arc e_*ij*_ is coming into *j* and is called an in-edge which represents that *j* is influenced by *i* (the mutation of *i* modifies the neighborhood of *j*, Figure 1C, nodes 2 and 3).

The procedure aggregates the information on the changes caused by all the nineteen mutations of an amino-acid position (Figures 1B,C). Namely, positions are mutated by the 19 remaining possible amino acid types, and in- and out-edges are aggregated for each position. This choice is made to reduce the complexity of the GCAT network in terms of number of links but also to synthesize the set of influences a position is visiting over its mutation by the 19 possible amino acid types. Positions that do not influence the system or are not influenced by the system are not represented in the GCAT network.

Because position amino acid neighborhoods and hence changes in position amino acid neighborhoods upon mutation are based on AANs built for a cutoff distance of 5 Å, the effect of the distance cutoff parameter on the GCAT network will deserve further investigation.

It should be noted that because mutations can change neighborhoods of amino acids that are not the chemical neighbors of the site of mutation, an edge in the GCAT network may connect nodes that are not chemical neighbors (they are neighbors neither in the WT AAN nor in the mutant’s AAN). In the toy-case of Figures 1A–C, the 1 →1′ mutation has caused a change in the neighborhood of nodes 2, 3 and 4 (B) compared to the WT AAN (A), and thus the edges from node 1 to nodes 2, 3, and 4 are added in the GCAT network (C). Please note that we do not add self-edges (e.g., the change of the neighborhood of node 1 caused by the mutation of itself) in the GCAT network. Similarly, the edges from node 2 to nodes 1 and 4 and from node 3 to nodes 1 and 2 are added in the GCAT network because the 2 → 2′ mutation causes a change in the neighborhoods of nodes 1 and 4 and the 3 → 3′ mutation causes a change in the neighborhoods of nodes 1 and 2. The 4 → 4’ mutation does not cause any change in neighborhoods compared to the WT. AAN, and thus no edges leave node 4 in the GCAT network (Figure 1C).

Node degree in directed network. In a directed network, the in-degree *k*_*in*_ of *i* is the number of neighbors of *i* connected to *i* by in-links (the link comes into *1*) and the out-degree *k*_*out*_ is the number of neighbors of *i* connected to *i* by out-links (the link leaves *i*).

GCAT classes- We classify the nodes (amino acid positions) in the GCAT network into four categories of influences based on their in- and out-degree (Figure 1E):

• Generate (G) category: nodes with k _*in*_ ≤ $\bar{k}$ _*in*_ and k _*out*_ > $\bar{k}$ _*out*_;
• Connect (C) category: nodes with k _*in*_ ≤ $\bar{k}$ _in_ and k _*out*_ ≤ $\bar{k}$ _*out*_;
• Absorb (A) category: nodes with k _*in*_ > $\bar{k}$ _in_ and k _*out*_ ≤ $\bar{k}$ _*out*_;
• Transmit (T) category: nodes with k _*in*_ > $\bar{k}$ _in_ and k _*out*_ > $\bar{k}$ _*out*_ *.*

With $\bar{k}$ _in_ the average of k _*in*_ over all the nodes of the GCAT network and $\bar{k}$ _*out*_ the average of k _*out*_ over all the nodes of the GCAT network. It must be noted that by definition $\bar{k}$ _in_ = $\bar{k}$ _*out*_ = $\bar{k}$ = |E|/N with |E| the number of edges and N the number of nodes, for any directed network.

Gephi- The Gephi software is used to visualize networks and compute network measures (Bastian et al., 2009). Gephi can be downloaded at https://gephi.org/users/download/. It has three windows: one working windows (overview) where the network can be manipulated and network measures computed, one data laboratory window where the data are available in a table format and a preview windows to look at the network. In Gephi, we used filters and statistics available on the upper right corner of the overview and node aspects, available on the upper left corner of the overview, to color the nodes according to attributes (sectors, functionally sensitive positions (FSP), surface exposed positions, *etc.*). The GCAT data and other information on the positions are available from the data laboratory (sectors, FSP, kin, k _out_, *etc.*). For more details on Gephy please see (Bastian et al., 2009). Below, we provide the definition of connected components, clustering coefficient and bidirectional edges, measures available in Gephi and used to analyze the GCAT network.

Connected component. Given a graph *G* = (*V, E*), a path *p* from *v* to *w* in G (*v* and *w* are vertices) is a sequence of vertices and edges leading from *v* to *w*. A connected component is a set of nodes connected to each other by paths. Given a directed graph, a strongly connected component (SCC) is a sub-graph of the original graph where all nodes are connected to each other by some path taking into account that some links can be followed in just one direction (Figure 1D). Gephi uses the Tarjan algorithm to compute connected components (Tarjan, 1972).

Clustering coefficient and triangular influences. The clustering coefficient (Watts-Strogatz), when applied to a single node, is a measure of how complete the neighborhood of a node is. That is the fraction of realized edges between neighbors of *i* and all possible edges between the neighbors of *i*. For a directed network, all possible edges, referred to as the theoretical number of edges between the neighbors of *i*, is computed as:

$$k_{i}x\left(k_{i}-1\right)$$ (1)

When applied to an entire network (Global CC), it is the average clustering coefficient over all of the nodes in the network. When an edge between two neighbors *j* and *k* of *i* is present, a triangle between *i*, *j* and *k* exists. The closer the CC is to 1, the more connected the neighbors of *i* are, the larger the number of triangles made between its neighbors and *i*. When there are no triangles between its neighbors and *i*, the CC is equaled to 0. We use CC to assess triangular influences between amino acids.

Bidirectional mutual influences. When the mutation of *i* changes the neighborhood of *j* and the mutation of *j* changes the neighborhood of *i*, it creates a bidirectional edge between the *i* and *j* positions, indicating mutual influences between the two positions and symmetrical mutational changes.

## Results

Our objective is to show the role of neighborhoods in the mutational response of amino acid positions. To do so, we build a tool that models the mutational influences between the amino acids of a protein from the impact of *in silico* mutations on the protein structure. This tool is then used to classify the amino acid positions according to categories of influences synonymous of position distinct mutation tolerances and hence of different position neighborhoods.

We chose 1PDZ 1BE9 as a case of study and generate using Fold X, the 2,185 PDB mutants that correspond to the 19 mutations of the 115 amino acids of 1BE9 (19 × 115 = 2,185), to have the complete mutational landscape of the 1BE9 protein structure. Each single mutation is produced changing only the side chain atoms at the site of mutation and keeping mutated structures with energy minima, hence viable and close to the WT structure. Precisely, neighbors of the mutation site are not allowed to move such that the differences between positions in the wild-type and in the mutated structures, are reduced to having new neighbors (e.g., side chain is longer), losing neighbors (e.g., side chain is shorter) and swapping neighbors (e.g., side chain has different spatial reach) (Figure 1F, upper and middle panels). This is to classify positions into positions with neighborhoods tolerating mutations, namely leading to less changes in the neighbors of positions upon mutations (Figure 1F, upper panel) and positions with neighborhoods not tolerating mutations, namely leading to more changes in the neighbors of positions upon mutation (Figure 1F, middle panel). If the neighbors are allowed to move, we cannot distinguish the class of positions that tolerates mutations without neighbor motions (Figure 1F, upper panel) from the class that tolerate mutations with neighbor motions (Figure 1F, lower panel). Yet the two classes are different because in the second case to accommodate the mutation the position of neighbors needs to be corrected. At this stage, we simply aim at determining two classes: one tolerating, one needing corrections.

The 1BE9 and its mutated version PDBs are modeled by amino acid networks (AAN) from which the neighbors of every position in the structures are computed based on the proximity of the atoms of the amino acids (Methods). The position neighborhoods of the WT AAN (Figure 1A) and of the 2,185 mutants AANs are compared to monitor neighborhood changes (Figure 1B). To model the neighborhood changes over the 19N mutation for each position, a network called GCAT is built with the following procedure (Detailed in Method) (Figure 1C). Amino acid positions are the nodes of the GCAT. When the mutation of an amino acid of type *a* at position *i* in the sequence modifies the neighborhood of a position *j,* an edge leaving *i* and coming to *j* is added between the two node positions (Figure 1B). This is an out-edge that represents the mutational influence of position *i* on position *j*. When the neighborhood of the position *i* is modified by mutations at position *j*, an edge leaving *j* and coming to *i* is added between the two positions (Figure 1B). This is an in-edge that represents the sensitivity of position *i* to mutation elsewhere (position *j* here). Arrows are added to a position *i* based on all the positions *j*s that change neighborhoods due to the 19 mutations at position *i* (all out-edges) and based on the changes in the position *i* neighborhood due to the 19 mutations of all the other positions (all in-edges) (Figures 1A–C). As a result, the nodes of the GCAT network are all the amino acid positions that change neighborhoods upon mutations and/or have their own neighborhood changed upon mutations elsewhere.

The GCAT nodes classify into four categories of influences based on their in-and out-degree, that is the number of in- and out-neighbors (Figure 1E) (Methods). The G category is for nodes with less in-edges than average but more out-edges than average, representing thus nodes which influence the system but are not much influenced by mutation elsewhere. The C category is for nodes with less in-edges and less out-edges than average, representing thus nodes with little influence on the system and seldom influenced by mutation elsewhere. The A category is for nodes with more in-edges and less out-edges than average, representing thus nodes with little influence on the system but often influenced by mutation elsewhere. The T category is for nodes with more in-edges and out-edges than average, representing thus nodes strongly influencing the system and strongly influenced by mutation elsewhere. The average degree used to sort nodes into GCAT categories is $\bar{k\;}$ = 7. The GCAT network for the 1BE9 protein is shown on Figure 1G with the amino acid nodes colored according to the four categories of position influences G, C, A and T and with the links colored based on the color of the node they leave.

The 1BE9 has 115 amino acids from positions 301 to 415, however the 1BE9 GCAT network has only 113 amino acid nodes because the positions 351 and 403 do not appear in the GCAT network. Neither position influences the system (their mutations modify no amino acid neighborhoods) or is influenced by the system (no change in their neighborhood upon mutation elsewhere). These are the only positions exhibiting mutational independency with all the other positions and complete tolerance to mutations at their positions and elsewhere. The 1BE9 GCAT has 21 nodes in the G category (18%), 56 in the C category (50%), 17 in the A category (15%) and 19 in the T category (17%) (Figure 1F).

Thus analyzing differences in the AAN neighborhoods resulting from *in silico* mutations while keeping a 1BE9 WT environment (*js* are not moved or mutated), reveals 4 position influence categories and establishes the role of neighborhoods in embedding the mutational response of amino acid positions. It also enables the classification of position neighborhoods according to mutation tolerance. The C and A positions have neighborhoods more tolerant to mutations at their positions (and elsewhere for the C positions) since mutations impact on average less position. In contrast, G and T positions have neighborhoods less tolerant to mutations at their positions (and elsewhere for the T positions) as mutations impact on average more position. G and T are categories where corrections through neighbor motions or neighbor mutations are needed for accommodating mutations.

Among the 17 amino acid types present in the 1BE9 sequence (there is no CYS, MET or TRP in 1BE9), some are not observed in the four mutational influence categories but most probably because the statistics is too small rather than because some amino acid types are unable to adopt some category. In fact, even with only 113 positions, the 4 topologies are reasonably distributed over the 17 types: three amino acid types appear in 4 categories (Gly, Pro and Arg), 10 in 3 topologies (Ala, Asp, Glu, Phe, Ile, Leu, Asn, Gln, Ser and Val), 3 in 2 (Lys, Thr and Tyr) and 2 in 1 category (Phe and His). The four topologies are adopted regardless amino acid sizes, chemical properties or spatial properties (Surface exposed *versus* buried positions). This signifies that no amino acid types, no amino acid properties or spatial positioning exclude an influence category and this agrees with a design of position mutational influences based on matching the amino acid position and neighborhoods features. We will have to investigate many more protein cases to look for the neighborhood features that associate with influence categories.

For now, we propose to determine whether the GCAT classes are related to three position properties, namely spatiality, functional sensitivity and sectors using Gephi to visualize the GCAT network and its features and to compute network measures.

We start by comparing the GCAT of surface exposed and buried positions (rASA property) because the response of surface exposed (SE) and buried positions to mutations are often found different (Halabi et al., 2009). But also because 76% of SE positions have less than 10 neighbors in the AAN against 6% of buried positions which could make SE positions more prompt to C influence category regardless neighborhood features, simply because the probability of influence is reduced due to having less neighbors. It is therefore important to check for potential bias related to the position spatiality before comparing the GCAT of functionally sensitive positions (FSP) with the GCAT of non-functionally sensitive positions and the GCAT of sectors with non-sector positions.

### Surface exposed and buried positions

**FIGURE 2:**
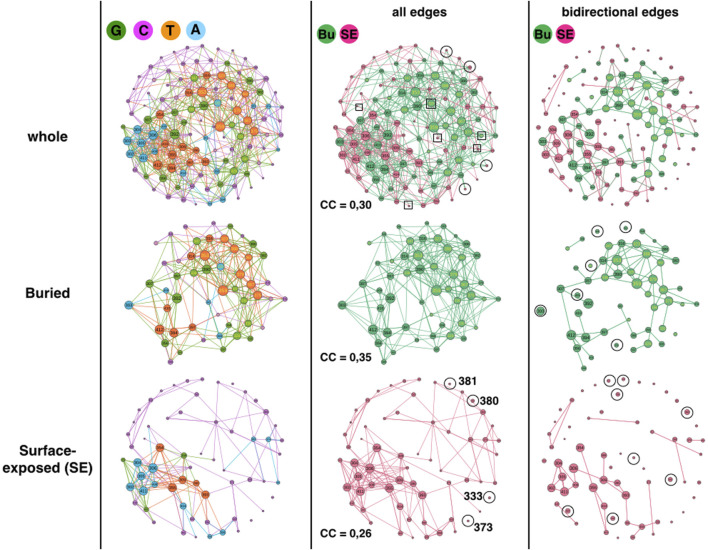
1BE9 GCAT network features of buried (Bu) and surface exposed (SE) sub-networks. The whole GCAT network, the buried and SE GCAT sub-networks are shown on the upper, middle and lower panels, respectively. The left, middle and right columns are the GCAT classification, all influences and bidirectional mutual influences, respectively. The square boxes indicate nodes that are involved in homogeneous influences only (i.e., edges SE to SE or edges Bu to Bu), the circles indicate nodes that are involved in heterogeneous influences only (i.e., edges SE to Bu or edges Bu to SE).

The first analysis compares surface exposed (SE) and buried positions (Figure 2). The GCAT is composed of 62 surface exposed amino acid nodes (55%) and 51 buried amino acid nodes (45%), numbers sufficiently similar to compare them without using percentages. The SE nodes partition in 3 G (5%), 42 C (68%), 12 A (19%) and 5 T (8%) while the buried nodes partition in 18 G (35%), 14 C (27%), 5 A (10%) and 14 T (27%) (Figure 2, left column). The first remark is on the percentage of the C category, which is not the percentage of positions with less than 10 neighbors in the AAN: 68% C category for SE positions against 76% with less than 10 neighbors and 27% C category for buried positions against 6% with less than 10 neighbors. Moreover, 50% of the SE positions in G, A and T categories, have less than 10 neighbors in the AAN. This means the number of neighbors in the AAN does not alone encodes the influence category. The SE and buried nodes appear in the four topologies, albeit to different percentages, indicating that the spatiality of a position in the protein structure does not encode a specific influence category either. Nevertheless, SE and buried positions adopt the influence categories with significant percentage differences. The A and C topologies are about twice more frequent for the SE positions whereas the G and T topologies are seven-times and three-times more frequent for buried positions, respectively. It therefore seems to us more reasonable to compare the GCAT of FSP and non-FSP and the GCAT of sectors and non-sectors only for buried positions.

Using strongly connected components (SCC), we compare the large-scale connectivity of the SE GCAT and the buried GCAT. SCC is a set of nodes connected to each other by paths taking into account the direction of the edges (Methods). The SE sub-network has 31 strongly connected components (SCC) against six for the buried sub-network. Our interpretation is that the mutations of positions that belong to the same SCC are potentially inter-dependent while the mutations of positions that belong to different SCC are independent. Multiple mutations within a SCC could amplify changes leading to more risk of damages or on the contrary correct changes leading to rescue mechanisms. Multiple mutations of positions from different SCC would produce neither amplification nor correction. The split of the SE positions in 31 SCC could be a mean to seclude surface areas and prevent propagation of mutational changes throughout the entire surface, contributing thus to the protein functional sustainability by limiting the impact of SE mutations. In comparison, less SCC could promote mutational influences propagating everywhere on the surface making the control of points of entrance to trigger function (ligand binding) more versatile to mutations. The possibility that many SCC limits the mutational sensitivity of SE positions is consistent with the result in (Reynolds et al., 2011), which shows that only eleven SE positions, spatially scattered (out of 39, in the paper they study 93 positions), have significant effects on ligand binding upon mutation.

The buried network has a less patchy network (6 SCC), which suggests propagation of mutational influences within most buried positions. Such propagation might bear alternative atomic motion paths that support functional allosteric mechanisms and contribute thus to functional robustness to mutations (Buchenberg et al., 2017). SE positions, except functional SE positions are less conserved than buried positions (Core of the protein) (Halabi et al., 2009), exhibiting a higher functional robustness to mutations that is consistent with collective influences scattered in patches as a mechanism to limit propagation of damages. Nevertheless, this is speculative and the difference in the SCC could simply be geometrical. The 51 buried amino acids are 3D-packed in the core of the protein while the SE amino acids are spread on the surface of the protein, making the distances between all the SE amino acids longer than between the buried amino acids. Hence having mutational influence paths connecting all the SE positions is more unlikely.

Considering all influences (i.e., all edges) and coloring the GCAT nodes according to their spatiality i.e., SE or buried (Figure 2, middle column), we can see that most nodes, buried (49 out 51 = 96%) and SE (54 out of 62 = 87%) combine mutational influences on buried and SE positions, exhibiting heterogeneous influences and limited independency between the SE and buried sub-networks. Only four SE nodes (320, 332, 384 and 402) have exclusive neighborhood influences on SE nodes and only two buried nodes (325, 376) have exclusive influences on buried nodes (Figure 2, middle column, upper row, square boxes). Four SE nodes (333, 373, 380 and 381) are isolated from the other SE nodes and are only influenced by buried nodes and only one (380) influences a buried position (375) (Figure 2, middle column, upper or lower rows, circles). No buried nodes are isolated from the other buried nodes. Thus, there are little exclusive influences whether homogeneous (SE-to-SE or buried-to-buried) or heterogeneous (SE-to-buried or buried-to-SE).

Considering triangular influences, i. e, three-position mutational influences, computed from the clustering coefficients (CC), we observe that there are slightly more triangular influences within the buried sub-network (CC = 0,35) than within the SE sub-network (CC = 0.26) (Methods). However, the CC of the whole network (CC = 0,30) is close to the CC averaged over the CCs of the SE and buried sub-networks ((0.35 + 0, 26)/2 = 0.305) indicating more heterogeneous triangular influences between SE and buried nodes than homogeneous triangular influences within the SE and the buried sub-networks, respectively (Figure 2, middle column, compare upper panel with middle and lower panel).

The heterogeneous influence between SE and buried nodes is also observed considering bidirectional influences (Figure 2, right column, compare upper panel with middle and lower panel). Bidirectional influences model pairwise mutual mutational influences (Methods). Out of 43 SE nodes involved in bidirectional edges (69%), twenty-six nodes are involved in heterogeneous bidirectional edges (Eighteen combined and eight exclusive SE-to-buried, 60%) and seventeen are exclusive SE-to-SE bidirectional edges (40%). Out of 50 buried nodes involved in bidirectional edges (98%), twenty-eight nodes are involved in heterogeneous bidirectional edges (Twenty-two combined and six exclusive buried-to-SE, 56%) and 22 are exclusive buried-to-buried bidirectional edges (44%).

In summary, the GCAT does not predict the SE and buried positions, the SE and buried sub-networks have different large-scale connectivity (SCC), the SE is broken in smaller patches compared to the buried sub-network and regardless the influences between all nodes, between three nodes or between two nodes, there are always influences between the nodes within each sub-network and between the nodes across the two sub-networks. This exhibits networks with some autonomy in the regulation of mutational influences and at the same time some coordination of influences between them.

### Sector and non-sector positions

**FIGURE 3:**
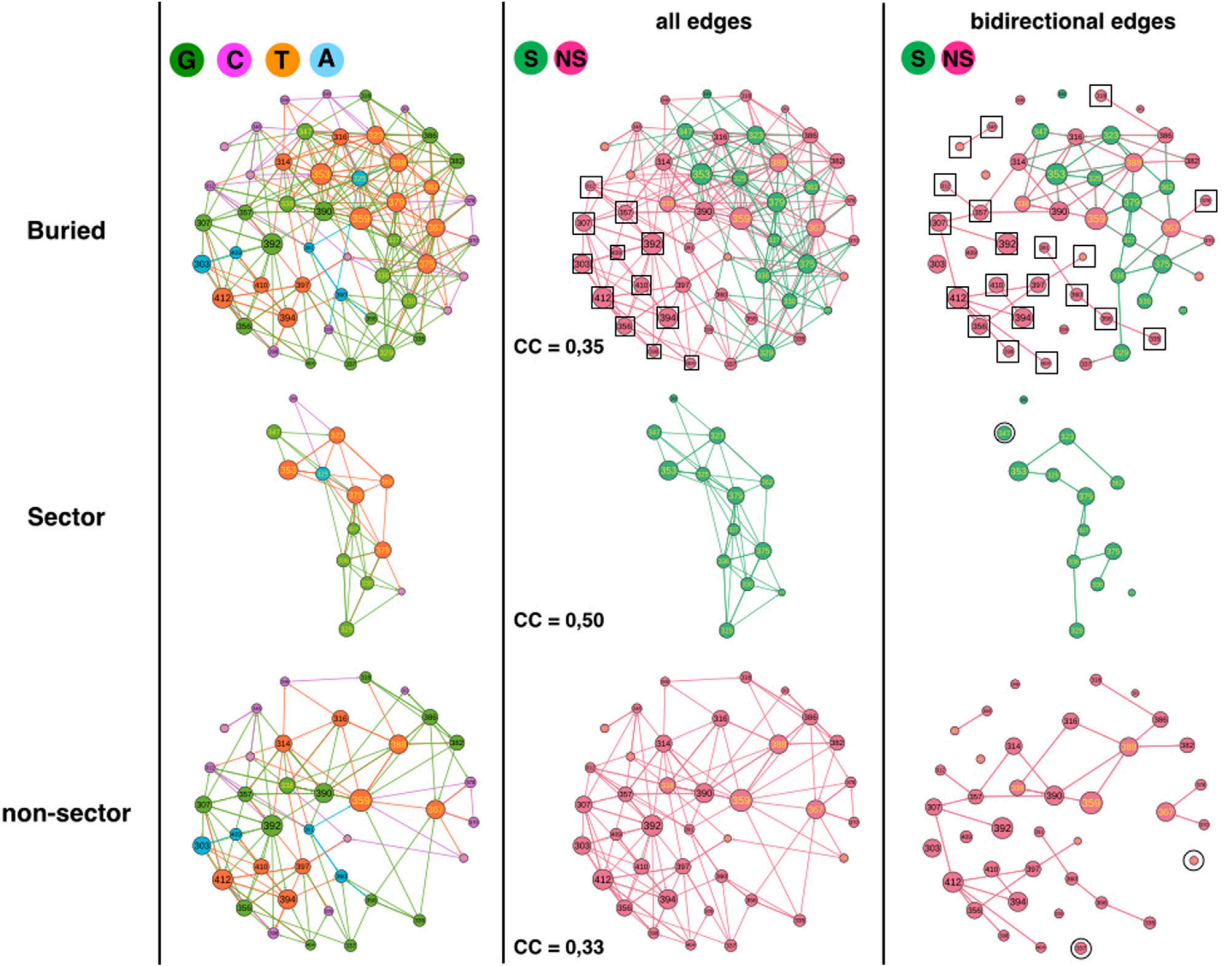
1BE9 GCAT network features of sector and non-sector sub-networks. The buried network, the sector and non-sector GCAT sub-networks are shown on the upper, middle and lower panels, respectively. The left, middle and right columns are the GCAT classification, all influences and bidirectional mutual influences, respectively. The square boxes indicate nodes that are involved in homogeneous influences only (i.e., edges sector to sector or edges non-sector to non-sector), the circles indicate nodes that are involved in heterogeneous influences only (i.e., edges sector to non-sector or edges non-sector to sector).

The second analysis compares sector and non-sector positions (Figure 3) (Halabi et al., 2009). The 51 buried positions split into 38 non-sector positions and 13 sector positions (out of 17 sector positions) such that the comparison requires extrapolation by a factor of about 3 or percentage comparison.

The sectors partition in 5 G (38%), 2 C (15%), 1 A (8%) and 5 T (39%) while non-sector positions partition in 13 G (34%), 12 C (32%), 4 A (10%) and 9 T (24%) (Figure 3, left column). Sector and non-sectors are distinguished by their percentage in the C category, twice more in the non-sectors and by their percentage in the T category, 1.6 times more in sectors. The sectors are sensitive to mutation elsewhere and influencing positions upon mutations (T positions) three times more often (T/C = 39/15 = 2.6) than they are not (C positions) which is consistent with their co-evolution relationships. In contrast, non-sectors are less often sensitive to mutation elsewhere and influencing positions than not (T/C = 24/32 = 0.75). Yet, clearly sectors and non-sectors visit the four influence categories, indicating that the GCAT classes do not distinguish the co-evolution relationships of sectors.

Sector and non-sector sub-networks have 4 and 8 SCC respectively, showing that sectors are slightly more sub-divided in patches. In fact, if the non-sector sub-network was as patchy as the sector sub-network, it should have 12 SCC and not 8 (i.e. 1.5 times more CC). We have no interpretation of the role of patches in the sectors but in (Halabi et al., 2009) the sectors also divided into two groups.

Considering all influences (all types of edges) and coloring the GCAT nodes in green if a sector position and in pink if a non-sector position (Figure 3, middle column), we can see that most nodes, sector (13 out 13) and non-sectors (26 out of 38 = 68%) combine mutational influences on sector and non-sector positions, exhibiting heterogeneous influences and limited independency between the two sub-networks. Twelve non-sector nodes (303, 307, 312, 356, 357, 392, 394, 398, 404, 409, 410 and 412) have exclusive neighborhood influences on non-sector nodes and no sector nodes influence exclusively sector nodes (Figure 3, middle column, upper panel, square boxes). There are neither sector nodes isolated from the other sector nodes (Figure 3, middle column, middle panel) nor non-sector nodes isolated from the other non-sector nodes (Figure 3, middle column, lower panel), indicating that there are no exclusive heterogeneous sector-to-non-sector or non-sector-to-sector influences. Thus, the sector nodes are all involved in heterogeneous combined influences while the non-sector positions have 32% of exclusive and homogeneous influences.

Considering triangular influences, we observe a higher fraction of nodes with triangular influences in the sector sub-network (CC = 0,50) than in the non-sector sub-network (CC = 0.33). The CC of the buried network (=0.35) is lower than the CC averaged over the sector and non-sector positions (0.42) indicating a lower number of heterogeneous triangles between sector and non-sector nodes than between SE and buried nodes.

The sector (11/13 = 85%) and non-sector (32/38 = 84%) sub-networks involve similar percentage of their nodes in bidirectional mutual influences (Figure 3, right column, compare upper panel with middle and lower panel). Out of 32 non-sector nodes involved in bidirectional edges, 13 are involved in heterogeneous bidirectional edges (11 combined and 2 exclusive non-sector-to-sector, 40%) and 20 are exclusive non-sector-to-non-sector bidirectional edges (60%) (Figure 3, right column, upper panel, squares). Thus, homogeneous non-sector-to-non-sector bidirectional influences dominate in the non-sector nodes. In contrast, out of 11 sector nodes involved in bidirectional edges, nine are involved in heterogeneous bidirectional edges (8 combined and 1 exclusive sector-to-non-sector, 82%) and 2 are exclusive sector-to-sector bidirectional edges (18%). Thus, it is heterogeneous sector-to-non-sector and combined bidirectional influences, which dominate in the sector nodes.

In summary, the GCAT does not predict the sector and non-sector positions, which have similar large-scale connectivity (SCC) and exhibit influences between all nodes, between three nodes or between two nodes within the sector (non-sector) sub-networks as well as across the two sub-networks. Yet, there are more three-node influences in the sectors nodes and less three-node heterogeneous influences between sector-to-non-sectors than between SE and buried nodes. The non-sector nodes have privileged bidirectional influences another feature not observed for the SE and buried sub-networks.

### FSP and non-FSP positions

**FIGURE 4:**
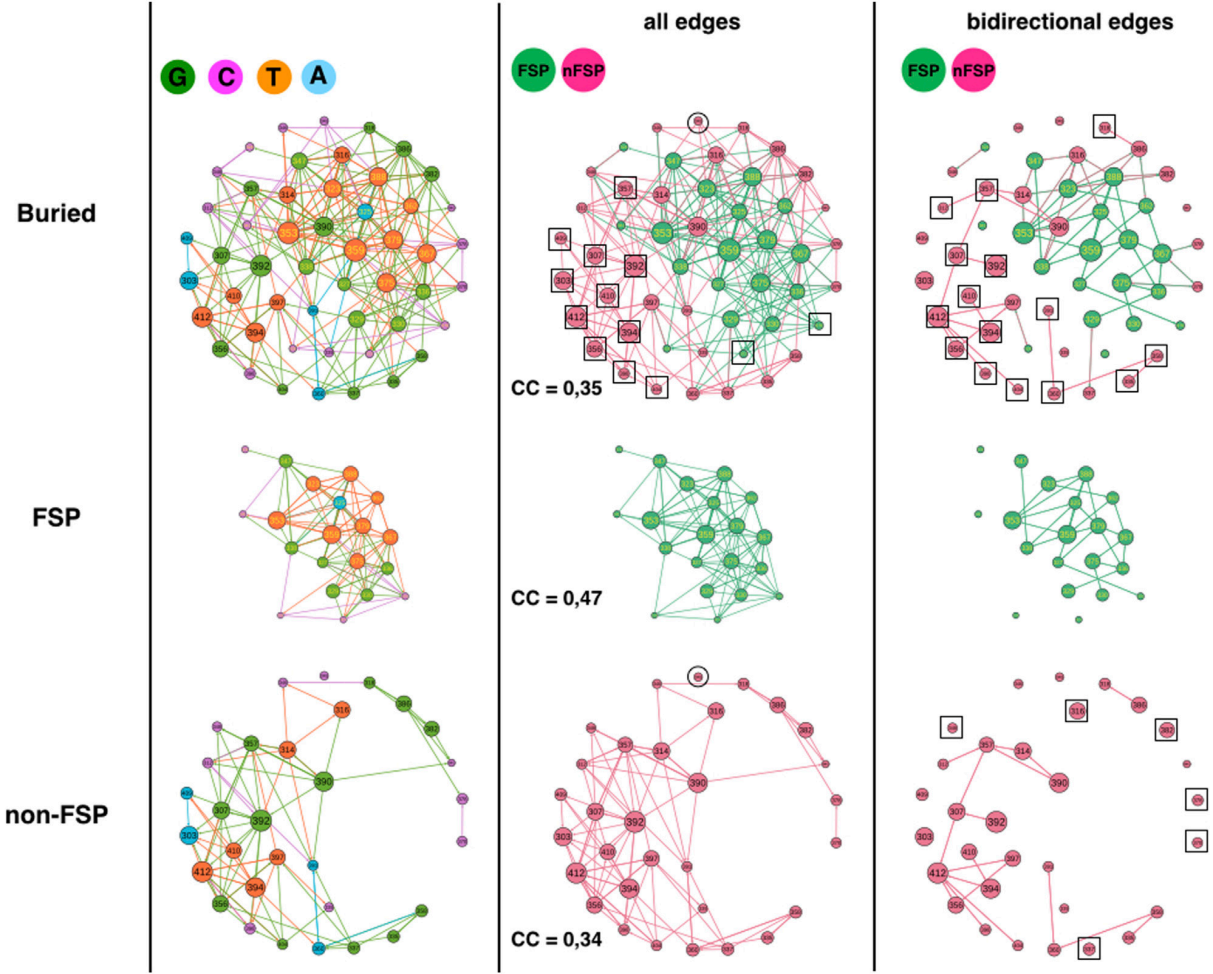
1BE9 GCAT network features of FSP and non-FSP sub-networks. The buried network, the FSP and non-FSP sub-networks are shown on the upper, middle and lower panels, respectively. The left, middle and right columns are the GCAT classification, all influences and bidirectional mutual influences, respectively. The square boxes indicate nodes that are involved in homogeneous influences only (i.e., edges FSP to FSP or edges non-FSP to non-FSP), the circles indicate nodes that are involved in heterogeneous influences only (i.e., edges FSP to non-FSP or edges non-FSP to FSP).

The third analysis compares FSP and non-FSP positions (Figure 4) (McLaughlin et al., 2012). The 51 buried positions split into 31 non-FSP positions and 20 FSP positions such that the comparison requires extrapolation by a factor of 1.6 or percentage comparison. The FSP partition in 6 G (30%), 5 C (25%), 1 A (5%) and 8 T (40%) while non-FSP positions partition in 12 G (39%), 9 C (29%), 4 A (13%) and 6 T (19%) (Figure 4, left column). 70% of the FSP adopt a category that influences system (G or T) and 80% of the FSP in A or C category, which influences the system less, are hotspots, i.e., positions that bind to the ligand of 1BE9 (5/6: 324, 372, 328, 376, 325). In contrast, only 29% of the FSP in G and T categories are hotspots. It must be noticed that T FSP are not more often sector (6 out of 8) than G FSP (5 out of 6). Assuming as a first approximation that the higher the number of arrows, the higher the likelihood of influences, it becomes consistent to have FSP adopting G and T categories, more out-edges increasing the likelihood of affecting neighbors and hence of impacting function. This assumption considers that the quantity of changes matters regardless the quality of changes. Binding to the ligand may make positions already more functionally susceptible to mutation requiring a C or A category to prevent systematic and irreversible functional failure upon mutation.

Along the same line, the non-FSP should be expected to have less out-edges and adopt preferentially C and A categories leading to functional tolerance to mutations. Yet, 58% of the non-FSPs adopt G or T category and 42% adopt A or C category, exhibiting only a slight difference with the FSP. Now, only 3 non-FSP positions are hotpots against nine hotspot FSPs, which may explain why non-FSP functionally tolerates the G and T categories. Now clearly, FSP and non-FSP adopt the four categories of influences, which suggest that FSP and non-FSP may differ by the quality of their changes rather than the quantity of changes.

However, one alternative, supported by the similarity of the G and C category percentages between the FSP and the non-FSP is that “faults” introduced by FSP mutations (G-type or C-type) must be mirrored by similar “correction types” introduced by non-FSP without functional consequences, to sustain adaptation and rescue mechanisms upon secondary site mutation. Under that hypothesis, both FSP and non-FSP must exhibit similar quantity of changes, i.e., appear in similar influence classes.

This hypothesis is consistent with non-FSP being about 3 times more often in the A category than FSP, exhibiting high sensitivity to mutation elsewhere with little influence on positions upon mutations (robust mutations) and twice less in the T category. The propensity for FSP to T category may correspond to positions capable of jeopardizing the function and be corrected (in-edges) compared to G FSP positions (less likely “repairable”).

The FSP sub-network has 2 SCC while the non-FSP sub-network has 15 SCC indicating a more patchy organization in the non-FSP sub-network. The FSP sub-network should have 10 SCC if it had the same disconnected feature than the non-FSP sub-network (i.e., 10-times more).

Considering all influences (all types of edges) and coloring the GCAT nodes in green if a FSP position and in pink if a non-FSP position (Figure 4, middle column), we can see that most nodes, FSP (18 out 20) and non-FSP (20 out of 31 = 64%) combine mutational influences on FSP and non-FSP positions, exhibiting heterogeneous influences and limited independency between the two sub-networks. Eleven non-FSP nodes (303, 307, 356, 357, 392, 394, 398, 404, 409, 410 and 412) have exclusive neighborhood influences on non-FSP nodes and two FSP nodes (372 and 376) influence exclusively FSP nodes (Figure 4, middle column, upper row, square boxes). There are no FSP nodes isolated from the other FSP nodes (Figure 4, middle column, middle panel) and only the non-FSP node 345 is isolated from the other non-FSP nodes, indicating it has exclusive hybrid non-FSP-to-FSP influences (Figure 4, middle column, lower or upper panel, circles). Thus, there are little exclusive homogeneous influences apart from within non-FSP positions (35%) and even less heterogeneous exclusive influences (3% for non-FSP).

The clustering coefficients (CC) of FSP and non-FSP positions are 0.47 and 0.34; respectively showing higher triangular influences in the FSP sub-network than in the non-FSP sub-network. The higher triangular influences of the FSP might be correlated with the sector feature since out of 20 FSP, 12 are sectors. This possibility is supported by the fact that the CCs of non-sector FSP and of sector-FSP are 0.38 and 0.51, respectively and the CC of non-FSP non-sectors is 0.35. The CC of non-FSP sector cannot be computed because only position 345 is non-FSP sector. The CC of the buried network (CC = 0.35) is lower than the CC averaged over the FSP and non-FSP positions (0.40) indicating a lower number of heterogeneous triangles between sector and non-sector nodes than between SE and buried nodes.

Considering bidirectional mutual influences, the FSP (18/20 = 90%) and non-FSP (25/31 = 81%), network involve similar percentage of nodes (Figure 3, right column, compare upper panel with middle and lower panel). Out of 25 non-FSP nodes involved in bidirectional edges, 10 are involved in heterogeneous bidirectional edges (4 combined and six exclusive non-FSP-to-FSP, 40%) and 15 are exclusive non-FSP-to-non-FSP bidirectional edges (60%) (Figure 4, right-column, upper row, squares). In contrast, out of 18 FSP nodes involved in bidirectional edges (90%), 11 are involved in heterogeneous bidirectional edges (9 combined and 2 exclusive FSP-to-non-FSP, 61%) and seven are exclusive FSP-to-FSP bidirectional edges (39%). Thus, it is heterogeneous bidirectional influences (FSP-to-non-FSP and combined), which dominate among FSP nodes but homogeneous non-FSP-to-non-FSP bidirectional influences among non-FSP nodes.

In summary, the GCAT does not predict the FSP and non-FSP positions but reveals some differences. FSP and non-FSP have distinct large-scale connectivity (SCC), the FSP have more three-node influences (higher CC) than non-FSPs, which on the contrary have more privileged bidirectional influences. This may indicate a link between influence motifs and impact of mutations.

## Discussion

The GCAT network is a tool that takes into account the asymmetry of mutational changes and allows visualizing the mutational influences of amino acid positions on one another. It is built from the comparison of the position neighborhoods in the amino acid network of the wild-type PDB 1BE9 and in the amino acid networks of *in silico* PDB mutants. The analysis of the GCAT at the level of individual nodes shows that 1BE9 positions split into four categories of influences called G, C, A and T. The positions G and T influence more other positions upon mutation than the position C and A while the positions A and T are influenced by the mutations of more positions than G and C. The different influence categories reveal AAN neighborhoods with different tolerance to mutations. The positions 301 and 415 are the only positions whose AAN neighborhoods tolerate all the mutations at their position and elsewhere. AAN neighborhoods of positions in the C category tolerate mutations at their position and elsewhere since they influence less than average other positions and are influenced by the mutation of less than average other positions. AAN neighborhoods of positions in the G category do not tolerate mutations at their position while neighborhoods of positions in the A category do not tolerate mutations elsewhere. AAN neighborhoods of positions in the T category do not tolerate mutations at their position and elsewhere. We end up with two classes of AAN neighborhoods, one class which tolerates more mutations than the other for which corrections through neighbor motions or neighbor mutations might be needed to accommodate mutations. Investigating what are the features of the neighborhoods for tolerance is future work requiring application of the GCAT on other proteins.

The GCAT does not predict the spatiality of positions, the sectors or the FSPs but its analysis through network measures highlights differences that generate hypothesis on mechanisms of tolerance and robustness to mutations (e.g., SCC numbers). We also observe different influence types with more triangular influences in FSP and sectors and a secluded bidirectional pairwise sub-network of influences in non-FSP and non-sectors. The CC and bidirectional edges were investigated looking for specific topologies of influences potentially embedding error-corrections or rescue mechanisms, co-evolution or adaptation through double or multiple mutations. From this point of view, the fact that the GCAT categories are adopted by sectors and non-sectors and by FSP and non-FSP introduces an alternative to looking for FSP or Sector-specificity. FSP and non-FSP sharing mutational influence characteristics makes possible that “mutational faults” introduced by FSP are mirrored by non-FSP “mutational corrections”.

Thus, the GCAT opens several perspectives towards understanding how positions cope with mutations and how to classify position tolerances and need for corrections. The next step is to repeat *in silico* mutations allowing neighbors to move, using *RosettaBackrub* for example (Smith and Kortemme, 2008; Lauck et al., 2010), and identify position neighborhoods corrected through motions, namely positions G or T in the present GCAT but C or A in the motion-allowed version. Position remaining in the G/T category even when motions is allowed would be a class where corrections might involve mutations of neighbors to tolerate mutations. Another perspective is to generate GCAT networks produced from AAN built on cutoff distances higher than 5Å to discriminate motion corrections involving chemical neighbors (cutoff at 5Å) and motion corrections involving neighbors above chemical reach, leading to a classification of multiple-scale corrections. We have previously shown using different cutoff, that positions in 1BE9 have different neighborhoods at different scales making multiple-scale corrections likely (Pacini et al., 2021). To improve our understanding of mutational influences and functional evolution, we could also investigate the GCAT of the PDB 1BFE, which is as 1BE9 but CRIPT-free.

## Conclusion

The GCAT models the complexity of positional influences that exists in proteins and offers some means to identify which positions influence which and in what terms with the perspective to improve our understanding of protein variants and their role on disease onset and drug treatment efficiency. At this early stage, the GCAT analysis is exploratory and aims at looking at influences from the scale of individual positions (GCAT category) to the scale of collective influences (connected components), considering influence types (all edges, triangular influences, bidirectional influences) and assessing independency between sub-networks of position features (heterogeneous *versus* homogeneous influences).

## Data Availability

The datasets presented in this study can be found in online repositories. The names of the repository/repositories and accession number(s) can be found below: https://github.com/lorpac/in_silico_mutations_analysis.
